# The Reserves of Beds in London Hospitals: King Edward's Hospital Fund for London Statistics

**Published:** 1915-05-15

**Authors:** 


### THE RESERVES OF BEDS IN LONDON HOSPITALS.

### King Edward's Hospital Fund for London Statistics.

Not the least interesting of the various tables of the Statistical Report is that giving the general particulars relating to the work and accommodation of 106 London hospitals for the year under review. In it the beds are divided under the headings, Medical, Surgical, Maternity, Paying, etc.; and this detailed information, interesting in itself, is useful in considering the financial section of the Report. The attempt to divide the out-patient cases into casualties, lying-in, and others, seems to have resulted unsatisfactorily; as the Report says, "the division . . . is based on figures supplied by the hospitals; but the varying proportions of casualties to others suggests that the definition of a casualty differs in different hospitals."

We give here a summary of the main features of the table:—

Beds, Days' Residence, Out-patients, and Attendances at 106 London Hospitals classified into Groups.

| Group | Beds: Total | Beds: Average Number in Daily Occupation | Average Number of Days each Patient was Resident | Total New Out-patients | Total Attendances | Average Number of Attendances per Out-patient |
|---|---|---|---|---|---|---|
| I. Larger General ... ... ... | 5,157 | 4,565·19 | 22·74 | 749,719 | 2,616,712 | 3.49 |
| II. Smaller General ... ... ... | 976 | 790·21 | 22 59 | 194,597 | 560,211 | 2·88 |
| III. Consumption ... ... ... | 588 | 543 | 63·55 | 15,720 | 84,749 | 5·39 |
| IV. Hospitals for Women ... ... | 284 | 259·43 | 22·72 | 19,430 | 76,826 | 3·95 |
| V. Children's ... ... ... ... | 688 | 619·79 | 24 55 | 127,095 | 399,326 | 3·14 |
| VI. Ophthalmic ... ... ... | 242 | 209·15 | 16·28 | 98,995 | 283,656 | 2·87 |
| VII. Hospitals for Epilepsy ... ... | 282 | 258·1 | 57·79 | 7,105 | 89,819 | 12·64 |
| VIII. Cottage ... ... ... ... | 317 | 217·69 | 23·08 | 5,218 | 26,459 | 5·07 |
| IX. Lying-in ... ... ... ... | 293 | 192·84 | 14·41 | 5 762 | 76,692 | 13·31 |
| Unclassified Hospitals ... ... ... | 1,708 | 1,379·40 | — | 91,895 | 423,642 | — |
| Totals ... ... ... ... ... | 10,535 | 9,034·71 | — | 1,315,536 | 4,638,092 | — |

It will be remembered that this table refers to 106 London hospitals only, but it is well known that the sole institution of considerable size not classified is St. Bartholomew's, where there was a daily occupation of about 566 out of 757 beds. We see, therefore, that out of 11,292 beds of the voluntary hospitals in London, 9,600 were in daily use in 1913. That means that 1,692, or approximately 15 per cent. of the whole, for one reason or another, were kept in reserve. In respect of the returns for 1914 and 1915 the percentage will be much smaller in view of the provision of beds for soldiers, and the efforts of hospitals to do the best possible for the civil population and for military patients. The figures for 1913, however, refer to normal conditions.

Fifteen per cent., at first sight, seems a broad enough margin to represent the reserve for emergency purposes, the temporary loss of accommodation through annual cleaning, or for other administrative reasons. In the general hospitals, where the average is about 12½ per cent., the system of periods of "taking in" for the different members of the staff, under which the beds of a physician or surgeon are gradually emptied in readiness for his turn for receiving during a certain number of days (when they will rapidly fill again), is one that for working purposes is very convenient, although it has the disadvantage of causing the unoccupied average to keep high. In these hospitals, also, particularly when the situation is an industrial district or near large railway stations, provision must always be made for the time when an accident causing a number of casualties will occur.

Hospitals for women and children, being in many respects general hospitals, must also provide a liberal margin; but in the special hospitals, where there are few, if any, casualties, and where the business of admission is more leisurely and the patients are resident for longer periods than elsewhere, the unoccupied margin should be very small indeed.

In the table given above it will be remarked that the heaviest unoccupied average is at the unclassified hospitals. We learn that at the London Fever Hospital, as an instance of one of the institutions included under the heading, in 1913 only 55 beds of the 160 were constantly used. This is quite understandable, for the hospital must be prepared at all times for a rush of patients; as there was no great wave of infectious fevers in 1913 the average number of beds occupied was low. Some other examples in the same group are:—

| | |
|---|---|
| Cancer Hospital ... ... ... ... | 89 of 102 |
| London Lock (Female) ... ... ... | 108 of 145 |
| Royal National Orthopædic ... ... | 176 of 181 |
| Cheyne Hospital for Children ... ... | 49 of 50 |
| Royal Waterloo (Children and Women) | 85 of 106 |
| St. Mary's, Plaistow (Women and Children) ... ... ... ... ... | 40 of 43 |

As with other matters referred to in the Statistical Report, we have often to draw deductions from figures alone, and figures do not help us to understand why the Cancer Hospital should be so much slower than the Orthopædic in admitting and discharging patients. Again, the remarkable divergence between the Royal Waterloo Hospital and the Plaistow Hospital for Women and Children stands out prominently.

There are cases where the full number of available beds were occupied practically every day of the year. One of these was Cheyne Hospital for Children (49 of 50) and St. Monica's Hospital for Children (23 of 24). In one of these institutions probably the majority of the patients were resident right through the year. (The average number of days each patient was resident were respectively 228 and 86.) In the other there was commendably good management in welcoming the coming guest very quickly after the parting guest was sped. But what of the annual cleaning? Was this done while the children were in the wards, or is there some part of the building where beds can temporarily be placed while the wards are closed?

Let us make no mistake: economical management of a hospital does not altogether lie in chaffering over the price of goods and extreme care in their use. It is true that unremitting watchfulness is requisite in these particulars, but there is a larger economy that is much more important. It lies in the regulation and disposition of the affairs of the institution in such a way that the utmost possible work shall be obtained from the machine of organisation.

We noted in a recent article that a particular hospital claimed that more in-patients could have been treated in a certain year with the addition only of an increase in the cost of provisions. This statement was not entirely accurate; the whole of the department Surgery and Dispensary would have to bear some extra expenditure; under Domestic, the sub-headings Renewal and Repair of Furniture, Bedding and Linen, Hardware and Crockery, Washing, would be affected. The charges allocated to other headings are so small and vague that they may safely be disregarded in an approximate calculation. But if we examine the whole of the expenditure side it will be found that much of the cost of the hospital will be the same if, within reasonable limits, regulated by special requirements and circumstances, more of the available beds are occupied, and that the statistical result will be a fall in the cost per occupied bed. It is evident, therefore, that the purposes of the truest economy will be best served by making the most of the institution and by treating every suitable patient that can properly be admitted to the wards. Here will the lay and medical element enter into friendly conflict. The resident medical man, generally busy, and probably at the present time overworked, will not be reluctant to delay filling beds for a few days. He knows each patient admitted requires a thorough overhauling, this means careful taking and recording of notes. On the administration side, however, there is the anxiety to make the most of the hospital and to produce material for favourable statistics. Each branch of the service has the good of the patient at heart, but does not look at the matter from the same point of view.

